# Pre-hospital intubation by anaesthesiologists in patients with severe trauma: an audit of a Norwegian helicopter emergency medical service

**DOI:** 10.1186/1757-7241-18-30

**Published:** 2010-06-14

**Authors:** Stephen JM Sollid, Hans Morten Lossius, Eldar Søreide

**Affiliations:** 1 Department of Research and Development, Norwegian Air Ambulance Foundation, Drøbak, Norway; 2 Department of Anaesthesiology and Intensive Care, Stavanger University Hospital, Stavanger, Norway; 3 Department of Surgical Sciences, Faculty of medicine, University of Bergen, Norway

## Abstract

**Background:**

Anaesthesiologists are airway management experts, which is one of the reasons why they serve as pre-hospital emergency physicians in many countries. However, limited data are available on the actual quality and safety of anaesthesiologist-managed pre-hospital endotracheal intubation (ETI). To explore whether the general indications for ETI are followed and what complications are recorded, we analysed the use of pre-hospital ETI in severely traumatised patients treated by anaesthesiologists in a Norwegian helicopter emergency medical service (HEMS).

**Methods:**

A retrospective audit of prospectively registered data concerning patients with trauma as the primary diagnosis and a National Committee on Aeronautics score of 4 - 7 during the period of 1994-2005 from a mixed rural/urban Norwegian HEMS was performed.

**Results:**

Among the 1255 cases identified, 238 successful pre-hospital ETIs out of 240 attempts were recorded (99.2% success rate). Furthermore, we identified 47 patients for whom ETI was performed immediately upon arrival to the emergency department (ED). This group represented 16% of all intubated patients. Of the ETIs performed in the ED, 43 patients had an initial Glasgow Coma Score (GCS) < 9. Compared to patients who underwent ETI in the ED, patients who underwent pre-hospital ETI had significantly lower median GCS (3 (3-6) vs. 6 (4-8)), lower revised trauma scores (RTS) (3.8 (1.8-5.9) vs. 5.0 (4.1-6.0)), longer mean scene times (23 ± 13 vs. 11 ± 11 min) and longer mean transport times (22 ± 16 vs. 13 ± 14 min). The audit also revealed that very few airway management complications had been recorded.

**Conclusions:**

We found a very high success rate of pre-hospital ETI and few recorded complications in the studied anaesthesiologist-manned HEMS. However, a substantial number of trauma patients were intubated first on arrival in the ED. This delay may represent a quality problem. Therefore, we believe that more studies are needed to clarify the reasons for and possible clinical consequences of the delayed ETIs.

## Background

Endotracheal intubation (ETI) is considered a key part of pre-hospital advanced life support (ALS) in critically ill and injured patients [1,2]. Recent studies [3-5] have, however, documented high failure rates and life-threatening complications with pre-hospital ETI. These high failure and complication rates have been linked to suboptimal airway management training and experience of the pre-hospital ALS provider [6]. To avoid these issues, some pre-hospital emergency medical systems (EMS), including the national helicopter emergency system (HEMS) in Norway, have used anaesthesiologists as pre-hospital emergency physicians for many decades [7-9]. However, anaesthesiologists active as pre-hospital emergency physicians regard pre-hospital airway management as challenging and recognise that such procedures likely warrant special training beyond the experience of in-hospital airway management [9].

Although there seems to be a general consensus on when pre-hospital ETI should be performed [1,2,10,11], limited data are available on the quality and safety of anaesthesiologist-managed pre-hospital ETI in trauma patients [8,12,13]. Furthermore, the extent to which indications for pre-hospital ETI are followed is not well documented. Therefore, we decided to perform an audit of pre-hospital ETI in seriously injured patients treated in a typical [7,8] Norwegian HEMS. We focused on whether trauma patients with an indication for pre-hospital ETI actually received it (quality of airway management) and whether ETI attempts were successful and without major complications (patient safety).

## Materials and methods

### Stavanger HEMS

The Stavanger HEMS is part of the national HEMS system of Norway, and its primary areas of operation are the mixed urban and rural districts of Rogaland County, which consist of just over 400,000 inhabitants. The medical conditions treated by the HEMS are approximately 2/3 non-traumatic and 1/3 traumatic [8]. In 2006, the Stavanger HEMS completed 1237 missions, of which 64% were by helicopter and 36% by rapid response vehicle (RRV) [9]. The RRV is used as a back-up when the helicopter cannot be used (due to weather conditions or other flight restrictions) or on missions close to the HEMS base. Both helicopter and RRV are operational day and night.

The HEMS crew consists of a pilot, a HEMS crewmember and a physician. The minimum requirement for HEMS physicians in Norway is 2 years of anaesthesiology as stated in a governmental report [9]. In addition flight operators require a flight operative initial training and checking. Pre-hospital ETI is performed at the discretion of the treating physician, and a variety of anaesthetic drugs are available to facilitate ETI. Written guidelines for pre-hospital ETI were available in the Stavanger HEMS during the study period and adhered to generally accepted indications for ETI outside the hospital [1,2,10,11]. Only minor adjustments were made to these guidelines during the study period. There was no specific difficult airway algorithm in the service in the period other than the one available in the anaesthesiology department under which it is organised. McCoy laryngoscope [2] and trans-tracheal kits were the only back up available in cases of difficult intubation until 2003 when the intubating laryngeal mask and the gum elastic bougie [2] were included.

All missions are recorded in a pre-hospital patient chart that includes core times (activation time, time of arrival, time of departure and time patient care is ended), vital parameters, patient data, applied interventions, drugs used and a brief summary of the mission. The charts also allow for scoring of the three components of the revised trauma score (RTS) [14]: systolic blood pressure, respiratory rate and Glasgow coma score (GCS) [15].

### Data collection

We retrospectively screened all records of patients treated by the Stavanger HEMS between 1994 and 2005 and extracted data from patients with severe trauma who either died before arriving at the hospital or were admitted to Stavanger University Hospital. We defined severe trauma as a primary diagnosis of traumatic injury and a National Committee on Aeronautics severity of injury or illness index (NACA) [16] (Table 1) score of 4 or higher.

**Table 1:** National Committee on Aeronautics severity of injury or illness index (NACA) [16]

Score	Definition
**0**	No injury or disease

**1**	Injuries/diseases without any need for acute physician care

**2**	Injuries/diseases requiring examination and therapy by a physician but hospital admission is not indicated

**3**	Injuries/diseases without acute threat to life, but requiring hospital admission

**4**	Injuries/diseases which can possibly lead to deterioration of vital signs

**5**	Injuries/diseases with acute threat to life

**6**	Injuries/diseases transported after successful resuscitation of vital signs

**7**	Lethal injuries or diseases (with or without resuscitation attempts)

We recorded data from the pre-hospital patient charts, as well as in-hospital data from the patient records. The data included the type of airway device and drugs used to facilitate ETI, complications and the use of monitoring, including capnography. We anonymised the involved HEMS physicians and recorded them as "anaesthesiologist specialist" or "resident". In cases in which the components of RTS were not scored, we retrospectively scored them based on data available from pre-hospital charts. RTS was then calculated based on a weighted formula [14].

### Ethics

The Regional Ethics Committee of Western Norway and the Norwegian Social Science Data Service approved the collection and recording of the study data.

### Statistics

Data were recorded into a database designed with File Maker (FileMaker Inc., Santa Clara, CA, USA). We used independent sample t-tests to compare mean values, the Mann-Whitney U Test to compare non-parametric median values and 2 × 2 tables with the chi-squared test for proportions. Mean values are presented with standard deviations and median values with the interquartile range. Statistics were computed using PASW Statistics 18 (SPSS Inc., Chicago, IL, USA). A p-value < 0.05 was considered statistically significant.

## Results

A total of 1255 cases matched our inclusion criteria for severe trauma. Table 2 shows the basic demographics. When comparing missions carried out by helicopter or RRV we found no significant difference in patient age, sex, NACA score, RTS or GCS. Further, mean time to scene and scene time were significantly shorter in RRV missions compared to helicopter missions: 9 ± 8 vs. 17 ± 10 min (p < 0.001) and 10 ± 8 vs. 20 ± 13 min (p = 0.001), respectively. There was no significant difference in transport times or the status of the treating physicians (anaesthesiologist specialist or resident).

**Table 2:** Basic demographics of the 12-year helicopter emergency medical service (HEMS) dataset (percentage calculated from total number of cases (n = 1255))

**Patient sex (n = 1253)**	930 male (74.1%)	322 female (25.7%)		

**Trauma category (n = 1255)**	1097 blunt (87.4%)	100 penetrating (8.0%)	55 other (4.6%)	

**NACA category (n = 1255)**	674 NACA 4 (53.7%)	361 NACA 5 (28.8%)	114 NACA 6 (9.1%)	106 NACA 7 (8.4%)

**RTS category (n = 1198)**	202 RTS < 4 (16.1%)	996 RTS > 4 (79.4%)		

**GCS category (n = 1194)**	353 GCS 3-8 (28.1%)	841 GCS 9-15 (67.0%)		

**Type of response (n = 1255)**	721 helicopter (57.5%)	534 RRV (42.5%)		

**Physician status (n = 1254)**	205 resident (16.3%)	1049 consultant (83.6%)		

GCS: Glasgow coma score

NACA: National Committee on Aeronautics severity of injury or illness index

RTS: Revised trauma score

HEMS: Helicopter emergency medical service

RRV: Rapid response vehicle

Among the 1255 cases, 240 (19%) intubation attempts were made pre-hospital with 238 recorded as successful, yielding a 99.2% success rate. Forty patients in this group died before arriving at the hospital and had a median GCS of 3 (3 - 3) and RTS of 0.0 (0.0 - 0.0). Additionally, 47 patients (16% of all intubated patients) were intubated immediately upon arrival in the ED (Table 3), all successfully. Among this group, 43 (92%) patients had an initial GCS lower than 9, of whom eight also had an initial RTS < 4 (Table 3). Patients who underwent attempted pre-hospital ETI had a significantly lower initial GCS, 3 (3 - 6) vs. 6 (4 - 8) (p < 0.001), and a lower initial RTS, 3.8 (1.8 - 5.9) vs. 5.0 (4.1 - 6.0) (p < 0.001), than those intubated in the ED. Significantly more patients who underwent attempted pre-hospital ETI also had both an RTS < 4 and a GCS of 3-8 compared to those intubated in the ED (56 vs. 17%, p < 0.001) (Table 3). Of the patients who underwent pre-hospital ETI, 71 were intubated without any drugs to facilitate ETI. Capnography use increased from 0% in 1998 to 79% in 2005 for successful pre-hospital ETIs (Table 4). Three of the pre-hospital ETIs were recorded with complications related to the procedure (Table 5).

**Table 3:** Distribution of patients according to National Committee on Aeronautics severity of injury or illness index (NACA) score, revised trauma score (RTS), Glasgow coma score (GCS) and transport mode

	NACA							RTS				GCS				RTS < 4 and GCS 3-8		RTS > 4 and GCS 9-15		Transport mode^§^			
	4		5		6		7		< 4		> 4		3-8		9-15						Air		Ground
Attempted pre-hospital endotracheal intubation (n = 240)	6		105		86		43^†^		134		105		207		28		131*		28		116		98
Intubated in emergency department (n = 47)	7		27		13				8		39		43		4		8		4		10		37
Not intubated (n = 968)	661		229		15		63^†^		63		852		103		809		60**		808		408		497

* 40 dead before arriving at hospital, ** 58 dead before arriving at hospital.

^† ^Three patients in the "Attempted pre-hospital endotracheal intubation" and five in the "Not intubated" group were scored as NACA 7 but were first declared dead after arrival at the hospital. These were not included in the "dead before arriving at hospital" group.

^§ ^Patients not transported from the scene by the service were not included in the table.

**Table 4:** Annual distribution of patients with severe trauma

Year	1994	1995	1996	1997	1998	1999	2000	2001	2002	2003	2004	2005	***Sum***
Number of patients with severe trauma*	80	105	112	83	78	83	114	109	126	134	122	109	*1255*
Arrived at hospital alive	73	93	103	74	74	79	101	96	117	128	112	99	*1149*
Fraction of patients dead before hospital arrival (%)	9	11	8	11	5	5	11	12	7	5	8	9	*9*
Attempted pre-hospital ETI	20	29	19	19	13	18	21	17	29	14	27	14	*240*
ETI in the emergency department	0	3	8	4	1	6	5	5	5	7	3	0	*47*
Fraction of ED ETI among total ETI (%)	0	9.4	29.6	17.4	7.1	25.0	19.2	22.7	14.7	33.3	10.0	0	*16.4*
Capnography used in pre-hospital ETI	0	0	0	0	0	1	0	1	3	4	14	11	*34*
Fraction of patients with pre-hospital ETI for which capnography was used (%)	0	0	0	0	0	6	0	6	10	29	52	79	*14*

*Defined as National Committee on Aeronautics severity of injury or illness index (NACA) score 4-7.

ETI: Endotracheal intubation.

ED: Emergency department.

**Table 5:** Cases of failed or complicated pre-hospital endotracheal intubation (ETI)

Case	ETI Successful	Type of injury	NACA	CGS	RTS	Outcome
Oesophageal intubation	Yes	Blunt	6	3	3.51	Dead < 24 h
Bleeding	No	Blunt	6	3	2.78	Dead < 24 h
> 2 ETI attempts	Yes	Blunt	6	5	5.03	Alive at discharge
Emergency tracheotomy	No	Burns with facial laceration	6	3	0.58	Dead < 24 h
Naso-tracheal intubation	Yes	Blunt	7	3	0.00	Dead on scene

NACA: National Committee on Aeronautics Severity of Injury or Illness Index

GCS: Glasgow coma score, RTS: revised trauma score.

There was no difference between the proportion of patients with pre-hospital ETI cared for by residents (13%) and consultants (88%) and the proportion of patients with ETI in the ED cared for by residents (13%) and consultants (87%) (p = 0.81). The individual physician performed between 1 and 11 (median 2) ETIs per year of the recorded pre-hospital ETIs. The total number of ETIs and the numbers of patients with attempted pre-hospital ETI and ETI in the ED varied from year to year but with no apparent temporal trend (Table 4).

Pre-hospital intubation attempts were more often made during helicopter missions than RRV missions (22 vs. 15%, p = 0.003). The mean scene time and transport time were significantly longer in patients with pre-hospital ETI compared to ETI in the ED: 23 ± 13 vs. 11 ± 11 min (p < 0.001) and 22 ± 16 vs. 13 ± 14 min (p = 0.001), respectively.

We found no difference in hospital days, ICU days or ventilator days between the two groups, but significantly more of the patients intubated in the ED were alive at discharge compared to those with attempted pre-hospital ETI (78 vs. 55%, p = 0.003).

## Discussion

In this audit of pre-hospital ETIs performed by anaesthesiologists in patients with severe trauma, we found a high success rate (99.2%) and few recorded complications. However, a substantial proportion of patients with an indication for pre-hospital ETI were not intubated until arrival in the ED.

The pre-hospital ETI success rate in patients with severe trauma was much better than those reported from many non-physician-staffed EMS systems [4,5] and similar to other physician-manned EMS systems [17,18]. The safety of pre-hospital ETIs should, therefore, not be a major concern. However, the overall quality of pre-hospital airway management is a different issue. We defined and measured quality as whether those with an indication for pre-hospital ETI actually received it. We found that, for example, 43 patients with an initial GCS on scene between 3-8 were intubated first on arrival in the ED. We could not identify a particular reason for this delay. In some of the pre-hospital patient charts, it was noted that ETI had been postponed due to short transport distance to the hospital. Indeed, the transport times were shorter in patients intubated in the ED. Additionally, their mean GCS and RTS were higher than in the patients intubated pre-hospital. Hence, the combination of short transport and a less severe injury may be put forward as a partial explanation for the finding.

Although we based our interpretation of the results on internationally accepted indications [1,2,10,11] for pre-hospital ETI in patients with severe trauma, there are several limitations to our audit. The data were collected retrospectively, which always entails some limitations in data quality. Future studies should collect data in a uniform manner to improve reliability and facilitate comparisons across systems and studies. The recently published Utstein style template for reporting data from pre-hospital advanced airway management [19] should be useful for this purpose. Future studies should also attempt to identify the reasons why HEMS physicians abstain from pre-hospital ETI in patients with severe trauma.

Our data were not adequately comprehensive to elucidate whether delayed ETI had any negative impact on outcome. On the contrary, the higher survival to discharge rate in the group first intubated in the ED could indicate a detrimental effect of pre-hospital ETI. Patients in the delayed ETI group were less severely injured (higher GCS and RTS) and more likely to survive than those intubated pre-hospital. Although this difference in injury severity may explain our findings, we believe that further studies are needed to clarify the clinical consequences of delaying ETI until arrival in the ED. A recent study from the Netherlands [20] also showed a failure to adhere to guidelines for pre-hospital ETI in traumatic brain injury in almost half of the studied population. Furthermore, the authors found a negative influence on respiratory and metabolic parameters in patients not intubated. Another recent study also indicated that delaying ALS in critically injured patients until arrival in the trauma centre worsens outcome [21].

One remaining question in this study is if any of the successful pre-hospital intubations were unnecessary or even harmful. We think this also must be considered a quality problem, but our data did not allow such an analysis. Still, 28 of the patients with pre-hospital ETI had both a RTS > 4 and GCS 9-15, which puts them in a category where the indication for ETI is unclear or at least signifies that other factors, besides severity of injury and GCS, must have influenced the decision to intubate. In the 40 patients with pre-hospital ETI who died before arriving at the hospital, we do not have data to document cause of death, but must assume, based on their low initial GCS and RTS, that death was related to their injuries and not any potential harm following ETI. Future studies on quality in pre-hospital ETI should investigate and address these issues.

Our audit was limited to one HEMS system, and the validity of our findings in other systems is, therefore, uncertain. However, our finding that a large proportion of patients with an indication for pre-hospital ETI did not receive it deserves further attention.

## Conclusions

This audit of pre-hospital ETI performed by anaesthesiologists in patients with severe trauma revealed that, despite a high success rate and few recorded airway management-related complications, a substantial number of patients with a pre-hospital indication for ETI were intubated only after arrival in the ED. Our audit did not fully uncover the reasons for this delay or determine whether the delay in ALS had detrimental consequences for patients. We believe that our audit indicates that future studies are needed and that a more standardised reporting system for pre-hospital advanced airway management would be useful for comparing airway management in different HEMS services.

## Competing interests

The authors declare that they have no competing interests.

## Authors' contributions

SJMS designed the study, collected the data, performed the statistical analysis and drafted the manuscript. HML and ES helped design the study and draft and review the manuscript. All authors have read and approved the final manuscript.
